# Clinical evaluation of fever-screening thermography: impact of consensus guidelines and facial measurement location

**DOI:** 10.1117/1.JBO.25.9.097002

**Published:** 2020-09-12

**Authors:** Yangling Zhou, Pejman Ghassemi, Michelle Chen, David McBride, Jon P. Casamento, T. Joshua Pfefer, Quanzeng Wang

**Affiliations:** a Food and Drug Administration, Center for Devices and Radiological Health, Silver Spring, Maryland, United States; b University of Maryland, Department of Mechanical Engineering, Baltimore County, Maryland, United States; c Johns Hopkins University, Department of Chemical and Biomolecular Engineering, Baltimore, Maryland, United States; d University of Maryland, University Health Center, College Park, Maryland, United States

**Keywords:** fever screening, thermography, medical guidelines, inner canthi, infectious disease epidemics, COVID-19, receiver operating characteristic (ROC) curve, Pearson correlation coefficients, thermometry, facial maximum temperatures

## Abstract

**Significance:** Infrared thermographs (IRTs) have been used for fever screening during infectious disease epidemics, including severe acute respiratory syndrome, Ebola virus disease, and coronavirus disease 2019 (COVID-19). Although IRTs have significant potential for human body temperature measurement, the literature indicates inconsistent diagnostic performance, possibly due to wide variations in implemented methodology. A standardized method for IRT fever screening was recently published, but there is a lack of clinical data demonstrating its impact on IRT performance.

**Aim:** Perform a clinical study to assess the diagnostic effectiveness of standardized IRT-based fever screening and evaluate the effect of facial measurement location.

**Approach:** We performed a clinical study of 596 subjects. Temperatures from 17 facial locations were extracted from thermal images and compared with oral thermometry. Statistical analyses included calculation of receiver operating characteristic (ROC) curves and area under the curve (AUC) values for detection of febrile subjects.

**Results:** Pearson correlation coefficients for IRT-based and reference (oral) temperatures were found to vary strongly with measurement location. Approaches based on maximum temperatures in either inner canthi or full-face regions indicated stronger discrimination ability than maximum forehead temperature (AUC values of 0.95 to 0.97 versus 0.86 to 0.87, respectively) and other specific facial locations. These values are markedly better than the vast majority of results found in prior human studies of IRT-based fever screening.

**Conclusion:** Our findings provide clinical confirmation of the utility of consensus approaches for fever screening, including the use of inner canthi temperatures, while also indicating that full-face maximum temperatures may provide an effective alternate approach.

## 1 Introduction

Fever is a key symptom of many infectious diseases that have caused epidemics, such as severe acute respiratory syndrome (SARS) in 2003, influenza A (H1N1) in 2009, Ebola virus disease (EVD) in 2014, and coronavirus disease 2019 (COVID-19).^1^^–^^6^ Fever screening is a medical countermeasure used at international borders, public transportation hubs, and hospitals to mitigate the propagation of these diseases. Often, a diagnostic based on radiative heat transfer from the human body [i.e., infrared (IR) thermometry] is used for primary screening in combination with other approaches, such as symptom questionnaires.^3^^,^^7^^,^^8^ If the subject is determined to be positive in primary screening, a secondary screening may be implemented including contact measurements (e.g., oral thermometry) and/or sampling for a laboratory test.

Noncontact infrared thermometers (NCITs)^9^^,^^10^ and infrared thermographs (IRTs)^11^ represent the primary device types currently used in practice for real-time screening of infectious disease during epidemics. NCITs and IRTs are passive remote sensing devices that detect mid- and/or long-wave IR radiation and convert that radiation to temperature based on the Stefan–Boltzmann law.^12^ NCITs estimate temperature at a reference body site (usually oral) based on measurements of a single region of skin (e.g., forehead),^13^ whereas IRTs provide a 2D temperature distribution—typically of the face—thus enabling a wider range of options for body temperature estimation. Although NCITs currently represent the primary tool for fever screening during epidemics,^14^ their accuracy has been called into question, particularly relative to IRTs.^15^^,^^16^ NCIT error may be due to a range of factors including the common use of forehead measurement locations, which are subject to fluctuations due to environmental factors such as ambient temperature and air flow.^7^

Human subject studies have demonstrated that IRTs can estimate body temperature and detect febrile individuals with moderately high accuracy. Several studies on IRT-based fever detection screening in hospital settings found “optimal” sensitivity (Se) and specificity (Sp) values—the point on the receiver operator characteristic (ROC) curve where both Se and Sp are high—in the 0.70 to 0.80 range.^15^^,^^17^^,^^18^ Hewlett et al.^19^ studied patients arriving at a hospital during the 2009 H1N1 influenza pandemic and found Se/Sp of 0.70/0.92 (AUC = 0.86) for IRT-identified subjects with fever above 100°F (37.8°C), although no data were provided on detection of subjects infected with H1N1 influenza. One study performed in an airport also indicated similar Se/Sp levels, yet identified a minimal number of infected travelers;^20^ this result was attributed to a lack of high fevers in the infected travelers identified. Similarly, in another airport screening study, Cho and Yoon^21^ were only able to detect six febrile travelers out of over 350,000 screened. This lack of Se may has been due in part to using a wide-field screening approach rather than the single-subject approach implemented in most of the aforementioned studies. In an extensive review of screening procedures during infectious disease epidemics, Mouchtouri et al.^22^ indicated that thermal diagnostics can be somewhat effective, yet often require great resource expenditures (e.g., device cost and personnel), and in the case of some epidemics such as SARS, their practical impact may be minimal. However, this review did not address variations in IRT device quality and implementation, which are likely significant factors in determining real-world effectiveness.^23^^,^^24^

Improvements in IRT-based temperature measurement accuracy could enable detection of lower-grade fevers (e.g., 37°C to 38°C). Such temperatures may be associated with early disease stages, such as when symptoms are starting to become evident in COVID-19^25^ and viral shedding is particularly high.^26^ This capability may be particularly relevant to transportation and containment, since individuals with fully developed symptoms may remain home or seek medical care, whereas those with less severe symptoms are more likely to travel. Furthermore, in diseases with significant person-to-person variations in symptom intensity, fully developed illness with low-grade fever would be more common. Alternately, enhanced IRT system accuracy could enable improved Se or Sp for high-grade fever, thus reducing the large number of false positives that are likely due to the high prevalence of afebrile individuals and wide variations in normal temperatures^27^ or slightly improving the likelihood of detected infected individuals.

Optimizing IRT-based screening requires consideration of fundamental device performance, implementation practices, and confounding factors such as environmental conditions. With the steady increase in the use of IRTs for medical applications, consensus documents for IRT evaluation and application have recently been published. International standard IEC 80601-2-59:2017^23^ provides recommendations for performance characterization of fever-screening IRTs. In a prior study, we implemented and evaluated these recommendations using two commercial IRTs,^28^ but we are not aware of any prior IRT clinical study that has implemented this standard. A consensus technical report, ISO/TR 13154:2017,^24^ describes best practices for IRT deployment, implementation, and operation. These include approaches that have not been commonly employed during disease epidemics, such as including a high-quality blackbody (BB) in the thermal image to minimize the impact of environmental factors, IRT instability, and drift. This report also recommends measurements be performed on individuals rather than a crowd, and that the inner canthus (tear duct) regions be measured to reduce measurement variability.

IRTs measure body surface temperatures that can be calibrated to or matched with corresponding oral or tympanic temperatures, which provide optimal discrimination based on ROC curve analysis. While some prior studies have equated oral and tympanic temperatures with core temperature, it is more accurate to say that these internal, yet accessible, sites act as well-correlated surrogates for core temperature.^27^^,^^29^ In thermal images, a variety of facial measurement locations and processing methods have been investigated to optimize IRT fever-screening performance. The inner canthi are thought to be an ideal location for noncontact temperature measurements. Perfused by the internal carotid artery, they have high temperature stability, are typically the warmest regions on the face, and have the highest correlation with internal body temperature.^11^^,^^30^^,^^31^ However, another study concluded that the correlation between ear (contact) and eye (noncontact) temperature was lower than expected.^32^ The maximum temperature around the eyes has also been studied.^8^ Several other IRT measurement regions have been evaluated, including the entire face,^1^^,^^19^^,^^33^ temples,^34^ nose,^34^ cheeks,^34^ ear,^34^ mouth (open and closed),^34^ and the forehead.^1^^,^^35^ From these previous studies, it is difficult to draw a clear conclusion regarding the optimal approach for temperature measurement from thermal images.

The purpose of this study was to generate independent data to assess the potential of IRTs for fever screening when implemented according to international consensus documents, while also elucidating the impact of facial measurement location and other key issues on IRT-based fever-screening performance. Specifically, our goals included: (a) acquisition of clinical IRT and reference temperature (oral temperature) data in a large population of febrile and afebrile subjects using standardized methods, (b) evaluation of facial measurement locations for their impact on correlation to—and absolute agreement with—reference temperature, and (c) comparative statistical analysis of febrile subject detection performance using these methods.

## 2 Methods

Over the course of 18 months—from November 2016 to May 2018—we conducted a clinical study of 596 subjects at the University Health Center of the University of Maryland (UMD) at College Park. Both Food and Drug Administration (FDA) and UMD Institutional Review Boards (IRBs) approved this study under FDA IRB study #16-011R and written informed consent was obtained from all subjects. All experiments were performed in accordance with relevant guidelines and regulations. Informed concern has been obtained for publishing recognizable images in this paper. The screening area was prepared according to consensus document specifications.^23^^,^^24^ Measurements of study subjects were performed with an oral thermometer, multiple NCITs, and two IRTs. The NCIT data and analysis will be the subject of a future paper. As detailed below, the current work focuses on analysis of a subset of the measurements acquired for each subject.

### 2.1 Experimental Setup and Thermograph Measurements

As noted in ISO/TR 13154^24^ and IEC 80601-2-59,^23^ a screening thermograph (ST) system includes an IRT and an external temperature reference source or BB. The reference source should have a known radiance temperature (function of real temperature and emissivity, ε) over the range of 33°C to 40°C with an expanded uncertainty (coverage factor of 2 for a ∼95% level of confidence) of ±0.3°C or less, and a combined stability and drift of ±0.1°C over the temperature interval for measurement, and its image size should be ≥20×20  pixels. A workable target plane (WTP) is a specific region of the target plane that is used for temperature measurement; it should accommodate a subject’s face positioning from 0.75 to 2.2 m above the floor. The WTP image pixel size should be at least 320×240. For ambient conditions, the temperature should be 20°C to 24°C and relative humidity should be 10% to 50%, based on ISO/TR 13154:2017. Forced cooling or heating of the target due to airflow or lighting should be avoided or at least minimized.

**Fig. 1:**
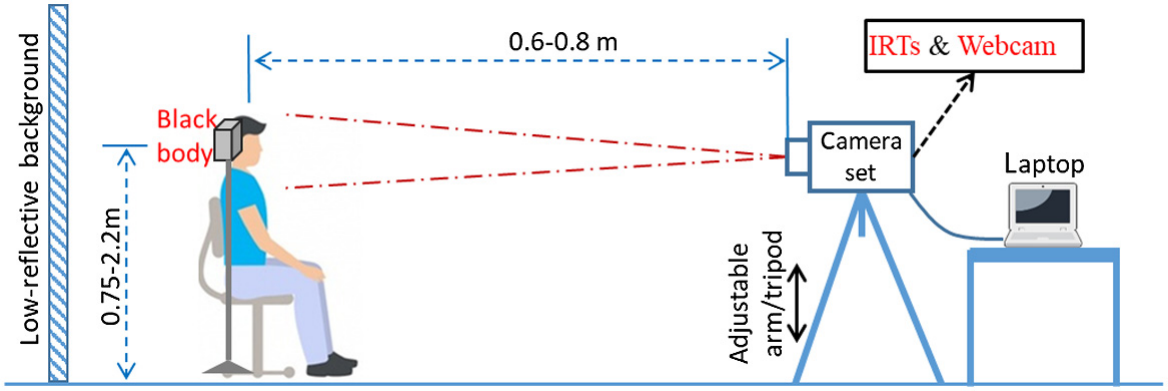
Diagram of the screening setup.

Based on ISO/TR 13154 recommendations, a screening setup was established as shown in Fig. 1. The setup consisted of a webcam (C920, Logitech, Lausanne, Switzerland) and two IRTs (IRT-1: 320×240  pixels, A325sc, FLIR Systems Inc., Nashua, NH and IRT-2: 640×512  pixels, 8640 P-series, Infrared Cameras Inc., Beaumont, TX) that were mounted in adjacent positions on a tripod. A WTP with dimensions of 320×240  pixels was identified for each IRT—the entire image for IRT-1 and a subset of the image in the most uniform region for IRT-2. We developed a graphical user interface with MATLAB to simultaneously control the webcam and two IRTs, and to collect images and patient/environmental information. Both IRTs had 30-Hz frame rates, and detailed specifications and fundamental performance testing results—including IRT measurement linearity with respect to a BB—can be found in our prior study.^28^ The laboratory accuracy of both IRTs with a BB satisfies the standard requirements (i.e., ≤0.5°C). A BB (SR-33, CI Systems Inc.) with a 4×4  in. emitter (image size≥20×20  pixels) was set at 35°C then positioned perpendicular to the sightline of the cameras and placed within the frame alongside the subject’s face for temperature drift compensation. We previously verified that BB drift and uncertainty satisfied relevant standards.^28^ The subject-to-camera distance was 0.6 to 0.8 m to ensure that both the subject’s face and the BB were included in the WTP with a resolution that satisfied recommended specifications (face image size≥240×180  pixels). Room temperature was maintained between 20°C and 24°C and relative humidity between 10% and 62%, as measured by a weather tracker (Kestrel 4500 NV, Weather Republic LLC, Downingtown, PA). The relative humidity was not exactly controlled within 10% to 50% as suggested by ISO/TR 13154:2017, with 7.5% of the subjects measured with relative humidity between 50% and 62%. Ambient temperature and subject ε (0.98 for human skin^36^) were entered into the IRT control program as input parameters for calibrating measured temperature. To prevent direct airflow to a subject’s face, an air vent in the room was blocked by a magnetic air deflector. A black, low-reflectivity cloth (ε=0.97; Type 822 E0.97, Group 8 technology, Provo, UT) was used as the backdrop.

**Fig. 2:**
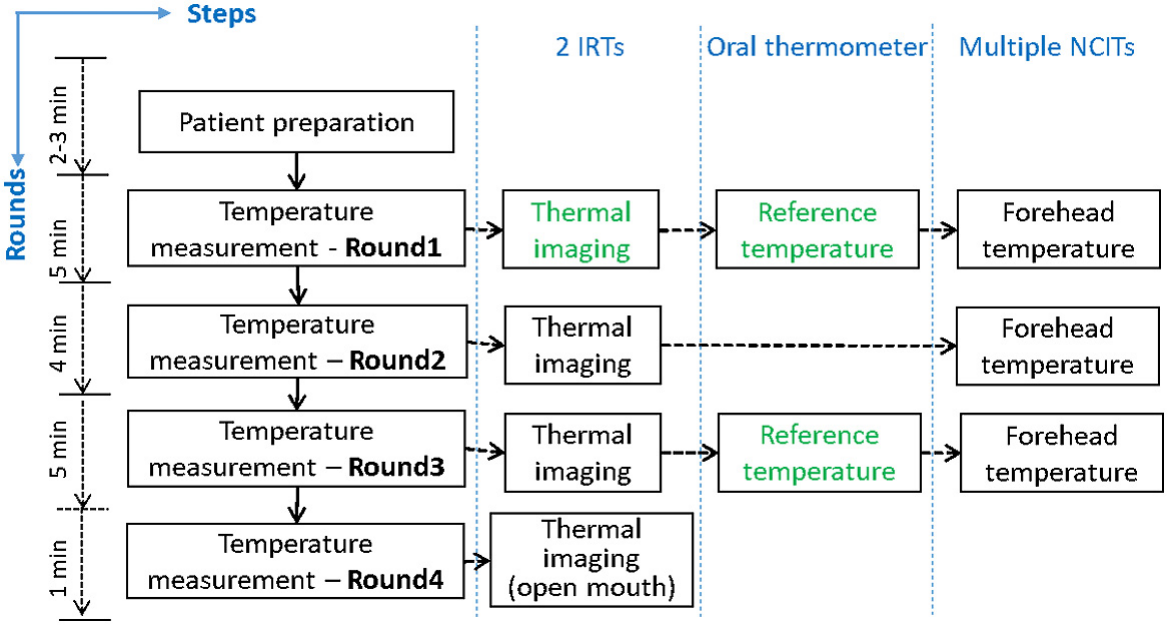
Flowchart of temperature measurement procedure—only data from the steps labeled in green were used in this study.

To minimize the influence of outside temperature, each subject was instructed to wait for 15 min before measurements started. Once seated, the subject was asked to remove all obstructions from the face (e.g., eyeglasses and hair on forehead) and look at the IRTs. Then, the tripod was adjusted to include the subject’s full face in the WTP. For each subject, four rounds of measurements were performed within ∼15  min following the procedure shown in Fig. 2. In each round, temperatures were measured with two IRTs (facial images) and several NCITs (forehead temperature). NCIT measurements performed in this study are beyond the scope of the current paper.

During each round of imaging, the webcam acquired one standard color image, while each IRT acquired three consecutive frames (acquisition time ∼0.1  s) that were averaged to reduce noise and form a single mean thermal image. In the last round, the subject was instructed to open their mouth to enable thermal imaging of sublingual tissue. Images from the second to fourth rounds of measurements were omitted in the current analysis, to better approximate a realistic screening scenario, but will be evaluated in a future study.

Oral thermometry was used to establish reference temperatures.^32^ A thermometer (SureTemp Plus 690, Welch Allyn, San Diego, CA) was placed under the subject’s tongue in a sublingual pocket (heat pocket). Then the temperature was read in two different modes, a “fast” mode in several seconds and a “monitor” mode after 3 min. We used the monitor mode data in this study since the monitor mode provides a superior accuracy compared to the fast mode. The monitor mode has accuracy of ±0.1°C, which was confirmed against a NIST-traceable contact thermometer (6413, Traceable^®^ Products, Webster, TX) using a laboratory water bath (89202-926, VWR International, Radnor, PA). The reference temperature ($T_{\mathrm{ref}}$) was calculated as the mean of two oral temperature measurements in monitor mode (during rounds 1 and 3). All subject data were discarded if the difference between two readings was larger than 0.5°C, due to the likelihood of a measurement error.

### 2.2 Facial Region Delineation and Temperature Calculations

**Fig. 3:**
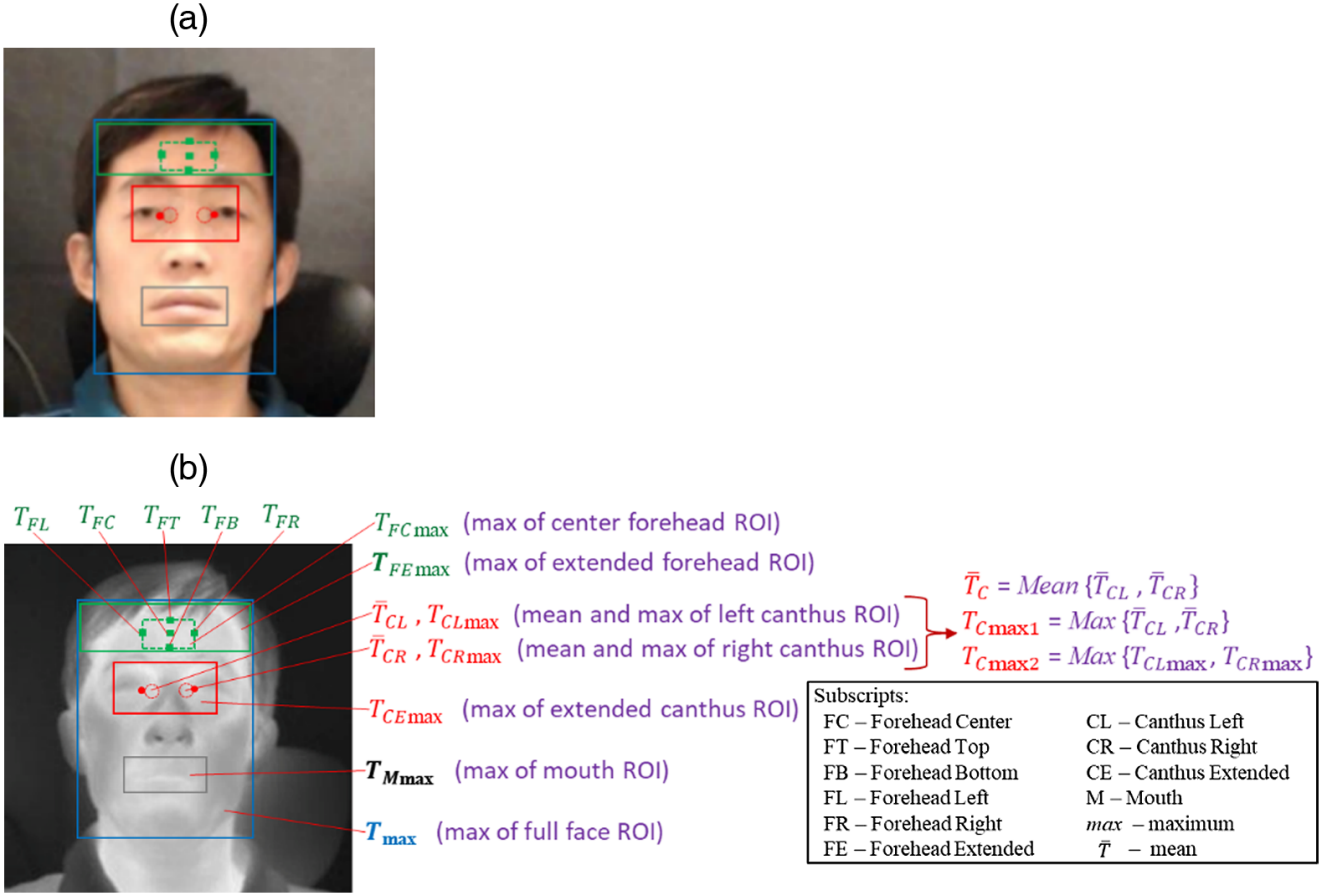
Delineated facial regions and critical points on (a) visible-light and (b) thermal images: forehead regions and points (green), canthi region and points (red), mouth region (gray rectangle), and entire face (blue rectangle). Photos are of author Q. Wang.

Temperatures from several facial areas—including the forehead, canthi, mouth, and entire face—were compared to assess impact on fever screening (Fig. 3). Standards documents do not specify a method for delineating facial key points such as canthi in thermal images, so we implemented an image registration approach^37^ to identify these points by matching facial landmarks on visible-light images to thermal images. However, key points for about half of these images required manual labeling. Based on the identified facial key points, different regions/points on thermal images were defined [Fig. 3(b)].

As shown in Fig. 3, the extended forehead area was determined in the vertical direction by hairline and eyebrows. The maximum single-pixel temperature in the extended forehead area ($T_{\mathrm{FEmax}}$) was first obtained. Then, the center forehead area was defined in the horizontal direction by the canthus points and in the vertical direction by 70% of the height of the forehead area from the bottom edge (eyebrows), and its maximum temperature (TFCmax) was obtained. Five points were defined in the forehead center region rectangle—the center point and the middle points of the top, bottom, left, and right edges. At each of these points, the mean temperature of a 3×3  pixel region was used to determine results ($T_{\mathrm{FC}}$, $T_{\mathrm{FT}}$, $T_{\mathrm{FB}}$, $T_{\mathrm{FL}}$, and $T_{\mathrm{FR}}$).

Two inner canthus points were identified in each thermal image using the registration method.^37^ Two small circular regions of interest (ROIs) were defined with a diameter of 13 pixels, using the inner canthus points as their outer edge. The mean and maximum temperatures of the left (${\overset{¯}{T}}_{\mathrm{CL}}$ and $T_{\mathrm{CLmax}}$) and right (${\overset{¯}{T}}_{\mathrm{CR}}$ and $T_{\mathrm{CRmax}}$) ROIs were obtained. From these values, the mean and maximum of ${\overset{¯}{T}}_{\mathrm{CL}}$ and ${\overset{¯}{T}}_{\mathrm{CR}}$ (${\overset{¯}{T}}_{C}$ and $T_{\mathrm{Cmax}1}$) and the maximum of $T_{\mathrm{CLmax}}$ and $T_{\mathrm{CRmax}}$ ($T_{\mathrm{Cmax}2}$) were also calculated. An extended canthus region was delineated, which was centered at the two canthus points and having a width of 96 pixels. Its top edge extended upward to the bottom of the eyebrows, and its bottom edge extended downward by the same amount. The maximum temperature ($T_{\mathrm{CEmax}}$) of this area was obtained.

The mouth region was defined by a rectangle that included all the facial key points around the mouth, then the maximum temperature of this region ($T_{\mathrm{Mmax}}$) was identified. The entire face region was defined horizontally by the edges of the face and in the vertical direction by the chin and hairline, and its maximum temperature was determined ($T_{\max}$).

### 2.3 Data Analysis

#### 2.3.1 Temperature compensation

Since IRTs exhibit varying degrees of instability and drift,^28^ a BB was used for thermal image compensation. By comparing the IRT-measured BB temperature ($T_{\mathrm{Bmeas}}$, averaged over the center area) with its set temperature ($T_{\mathrm{Bset}}$) of 35°C, we identified an appropriate offset for each image. Specifically, the equation $T_{\mathrm{off}}=T_{\mathrm{Bset}}-T_{\mathrm{Bmeas}}$ was used, where $T_{\mathrm{off}}$ is the offset value added to every pixel in the image. Unless otherwise specified, all data in this paper were compensated with the BB in this manner.

#### 2.3.2 Statistical analysis

To assess the effect of facial location for fever screening, results were analyzed using comparative boxplots, scatter plots, Pearson correlation coefficients (r value), and receiver operating characteristic (ROC) curves. Temperatures obtained from thermal images were compared with the reference temperature ($T_{\mathrm{ref}}$). The pairwise difference between $T_{\mathrm{ref}}$ and temperatures extracted from different facial locations ($T_{\mathrm{IRT}}$) was obtained. Comparative boxplots were used to display and compare pairwise differences. The r was used to quantify the degree of linear correlation between $T_{\mathrm{IRT}}$ and $T_{\mathrm{ref}}$.

ROC curves^38^—which plot Se (true positive rate) versus 1-Sp (true negative rate) for a range of cutoff levels—were used to assess discrimination between febrile and afebrile subjects. True febrile status was defined as $T_{\mathrm{ref}}\geq 37.5{}^{\circ}C$.^20^^,^^39^^,^^40^ An ROC curve for each facial temperature location was generated from 1000 IRT-based cutoff temperatures equally spaced between 30°C and 40°C. At each cutoff temperature, the numbers of positive and negative subjects—and thus pairs of Se/Sp values—were determined based on true febrile status. The area under the ROC curve (AUC) was calculated to provide an aggregate measure of performance (where an AUC of 1 indicates perfect diagnostic performance). AUC values for different facial temperatures were compared using pairwise tests with a 95% confidence interval (Analyse-it, Method validation edition, Analyse-it Software, Ltd., Leeds, UK). For each ROC curve, the optimal IRT cutoff temperature was determined as either the point on the ROC curve closest to (0, 1)^38^ or the Youden index.^41^ As both methods yielded very similar results, we only used the former method to find the optimal cutoff temperature which minimizes the quantity $[{\left(1-\mathrm{Se}\right)}^{2}+{\left(1-\mathrm{Sp}\right)}^{2}$] and thus yields the greatest combined Se and Sp.

## 3 Results

### 3.1 Subject Demographics

A total of 596 subjects were recruited; all were at least 18 years old and free of disease affecting the skin in canthi area or forehead one week prior to the screening date. Among these subjects, 33 had two oral temperature readings with difference >0.5°C, and thus were removed from the database. Of the remaining 563 sets of subject data, we excluded 19 from IRT-1 and 23 from IRT-2 due to motion artifacts that degraded image quality. Finally, we had 544 data sets for IRT-1 and 540 data sets for IRT-2. These data sets will be released to the public in the near future. Demographic information for study subjects is summarized in Table 1.

**Table 1:** Demographics of study subjects.

		IRT-1		IRT-2	
		Subjects	%	Subjects	%
	Female	329	60.5	328	60.7
	Male	215	39.5	212	39.3
Age	18 to 20	263	48.3	262	48.5
	21 to 30	247	45.4	244	45.2
	31 to 40	21	3.9	21	3.9
	41 to 50	4	0.7	4	0.7
	51 to 60	7	1.3	7	1.3
	>60	2	0.4	2	0.4
Ethnicity	White	257	47.2	254	47.0
	Black/African-American	78	14.3	79	14.6
	Hispanic/Latino	39	7.2	39	7.2
	Asian	138	25.4	136	25.2
	Multiracial	30	5.5	30	5.6
	American Indian	2	0.4	2	0.4
$T_{\mathrm{ref}}>37.5{}^{\circ}C$		47	8.6	47	8.7

### 3.2 Temperature Measurement Accuracy and Correlation

**Fig. 4:**
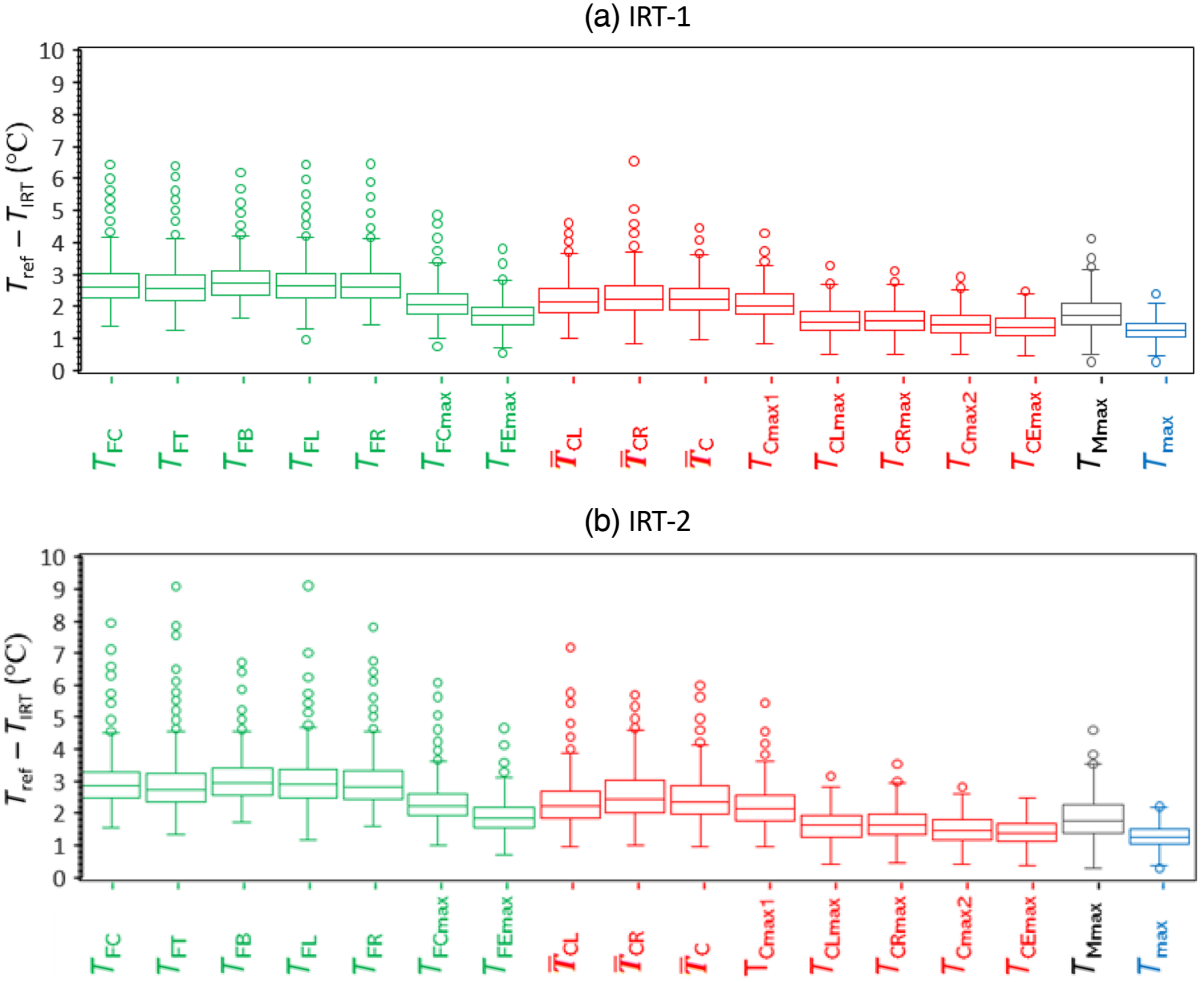
Boxplots of pairwise differences between $T_{\mathrm{IRT}}$ and $T_{\mathrm{ref}}$. The parameter $T_{\mathrm{IRT}}$ on the y axis represents all the IRT-measured facial temperatures on the x axis.

Temperatures for different facial locations from thermal images were compared with $T_{\mathrm{ref}}$. For each IRT, comparative boxplots of pairwise differences with $T_{\mathrm{ref}}$ were constructed (Fig. 4). For all temperatures from both IRTs, pairwise differences were positive. This is consistent with prior studies, and expected given that sublingual tissue is not typically subjected to the levels of convective heat loss that external tissues experience.^42^ Overall, the forehead region showed the greatest discrepancy, then the canthus regions, and the entire face maximum shows the least difference.

Pearson correlation coefficients for all measurement locations were highly consistent with the pairwise difference results (Table 2). Among the seven forehead temperatures, r values varied from 0.39 to 0.63 with $T_{\mathrm{FEmax}}$ showing the highest correlation and agreement with $T_{\mathrm{ref}}$, followed by $T_{\mathrm{FCmax}}$. The maximum temperature around the mouth ($T_{\mathrm{Mmax}}$) showed results that were similar to $T_{\mathrm{FEmax}}$. Correlation results for inner canthi temperatures were in general higher than for the forehead, ranging from 0.51 to 0.76. The highest r values (>0.7) were found for maximum temperatures across left and right inner canthi regions ($T_{\mathrm{Cmax}2}$), the extended inner canthi region ($T_{\mathrm{CEmax}}$), and the entire face ($T_{\max}$). As $T_{\max}$ had the largest r value, this metric may best estimate $T_{\mathrm{ref}}$.

**Table 2:** Pearson correlation coefficients (r values) between facial temperatures and $T_{\mathrm{ref}}$.

	Forehead							Inner canthi								Mouth	Face
	$T_{\mathrm{FC}}$	$T_{\mathrm{FT}}$	$T_{\mathrm{FB}}$	$T_{\mathrm{FL}}$	$T_{\mathrm{FR}}$	$T_{\mathrm{FCmax}}$	$T_{\mathrm{FEmax}}$	${\overset{¯}{T}}_{\mathrm{CL}}$	${\overset{¯}{T}}_{\mathrm{CR}}$	${\overset{¯}{T}}_{C}$	$T_{\mathrm{Cmax}1}$	$T_{\mathrm{CLmax}}$	$T_{\mathrm{CRmax}}$	$T_{\mathrm{Cmax}2}$	$T_{\mathrm{CEmax}}$	$T_{\mathrm{Mmax}}$	$T_{\max}$
IRT-1	0.46	0.41	0.49	0.47	0.43	0.55	0.63	0.60	0.58	0.63	0.65	0.70	0.71	0.73	0.75	0.60	0.78
IRT-2	0.46	0.39	0.49	0.46	0.41	0.54	0.62	0.53	0.51	0.56	0.59	0.70	0.69	0.73	0.76	0.60	0.79

**Fig. 5:**
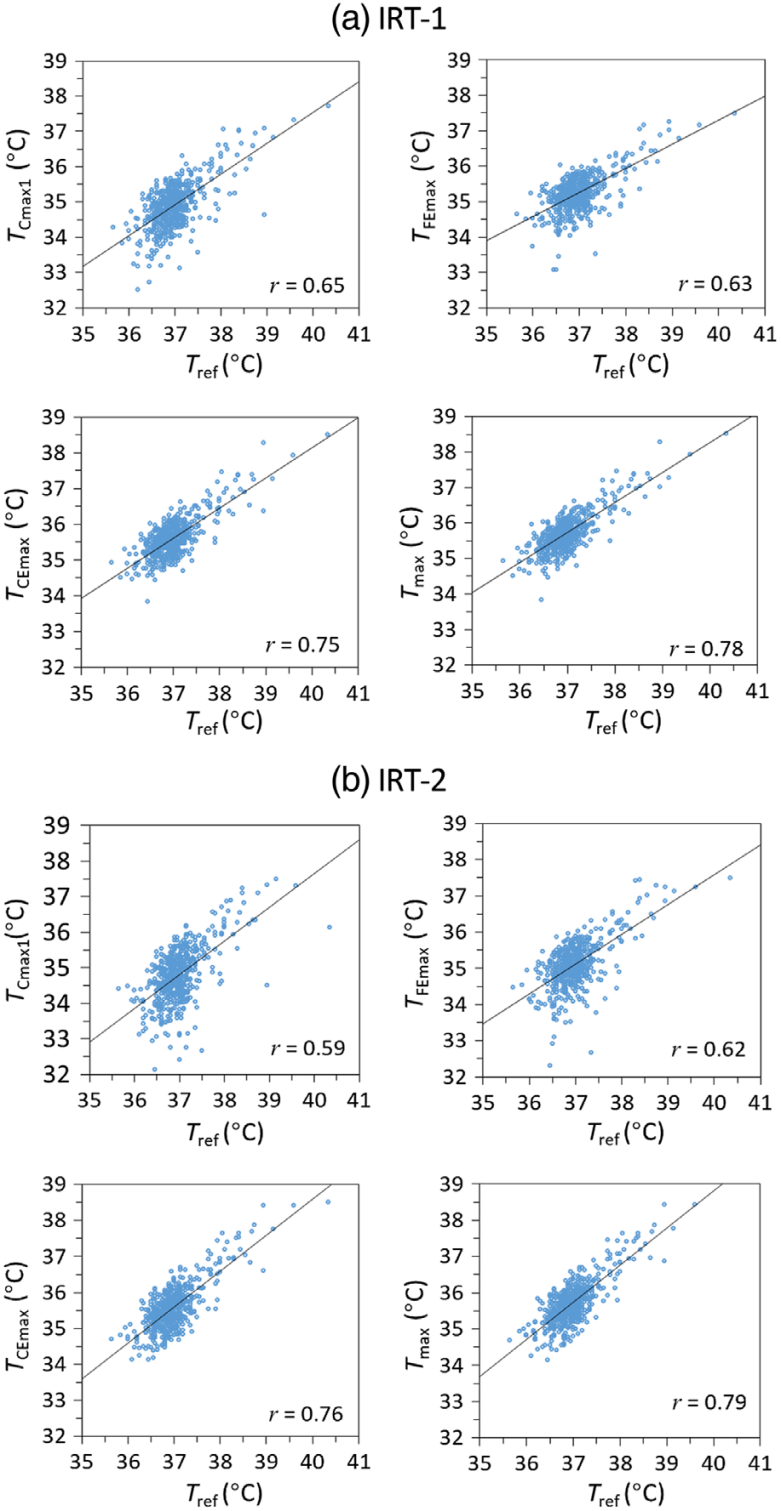
Scatter plots of $T_{C\max1}$, $T_{\mathrm{FEmax}}$, $T_{\mathrm{CEmax}}$, and $T_{\max}$ versus $T_{\mathrm{ref}}$, with linear fits and correlation coefficients. (a) IRT-1 and (b) IRT-2.

Facial temperature correlations were further analyzed with scatter plots of $T_{\mathrm{IRT}}$ versus $T_{\mathrm{ref}}$ for selected facial locations (Fig. 5). These included $T_{\mathrm{Cmax}1}$, which aligns with ISO/TR 13154 recommendations (although this approach is not explicitly defined), as well as $T_{\mathrm{FEmax}}$, $T_{\mathrm{CEmax}}$, and $T_{\max}$, which showed the best r values for extended forehead, extended inner canthi, and whole face regions, respectively. As expected, scatter plots show a large group of $T_{\mathrm{ref}}$ values near 37°C, representing the high proportion of afebrile subjects (84% of $T_{\mathrm{ref}}$ readings were between 36.4°C and 37.4°C). $T_{\mathrm{Cmax}1}$ results showed the greatest variability, most notably in the subfebrile range. $T_{\mathrm{FEmax}}$ results showed improved correlation with $T_{\mathrm{ref}}$ in this range, as well as across higher $T_{\mathrm{ref}}$ values. $T_{\mathrm{CEmax}}$ and $T_{\max}$ measurements were less variable than other measurements and well-correlated to $T_{\mathrm{ref}}$, approaching a linear relationship. Data for the two IRT systems showed only minor differences in correlation.

### 3.3 Fever Detection Performance

**Fig. 6:**
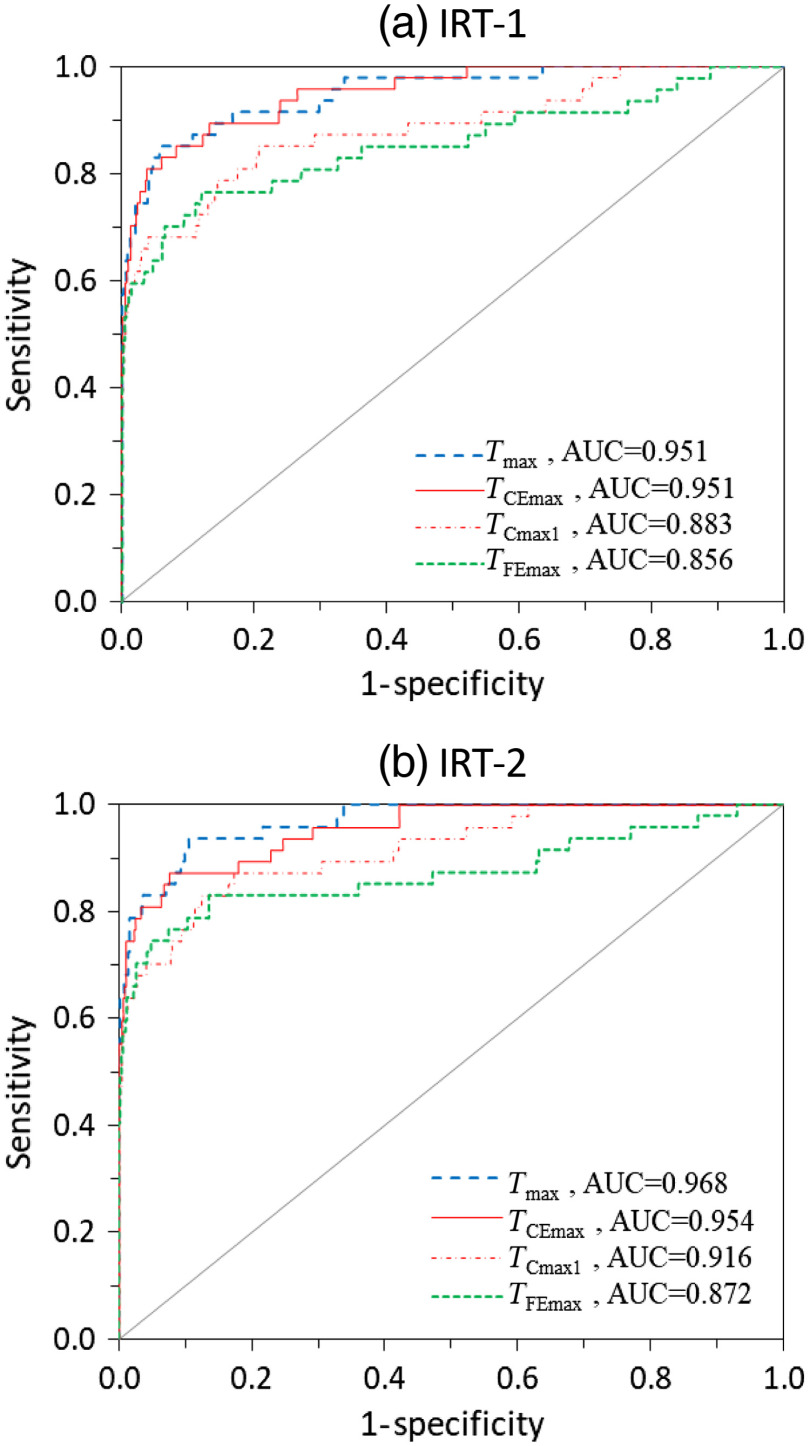
ROC curves for febrile subject ($T_{\mathrm{ref}}\geq 37.5{}^{\circ}C$) detection with (a) IRT-1 and (b) IRT-2 using facial temperatures of $T_{\max}$, $T_{\mathrm{CEmax}}$, $T_{C\max1}$, and $T_{\mathrm{FEmax}}$.

ROC curve analysis was used to evaluate performance of IRT-based detection of febrile subjects and its dependence on facial measurement location. Figure 6 shows ROC curves for $T_{\mathrm{Cmax}1}$, $T_{\mathrm{FEmax}}$, $T_{\mathrm{CEmax}}$, and $T_{\max}$, while AUC values for all measurements are found in Table 3. In Fig. 6, the y=x line denotes random discrimination; all ROC curves from our study were well above this line. $T_{\max}$ and $T_{\mathrm{CEmax}}$ yielded optimal ROC curves with AUC values >0.95, indicating high discrimination effectiveness. IRT-2 showed slightly better performance for $T_{\max}$ than $T_{\mathrm{CEmax}}$. ROC curves for $T_{\mathrm{Cmax}1}$ and $T_{\mathrm{FEmax}}$ indicated lower performance, with the most notable feature being a slow convergence to high Se as Sp decreased. In general, discrimination performance for $T_{\mathrm{IRT}}$ aligned well with correlations to $T_{\mathrm{ref}}$, as shown in Table 2.

**Table 3:** AUC values from the ROC curves of different facial temperatures.

	Forehead							Inner canthi								Mouth	Face
	$T_{\mathrm{FC}}$	$T_{\mathrm{FT}}$	$T_{\mathrm{FB}}$	$T_{\mathrm{FL}}$	$T_{\mathrm{FR}}$	$T_{\mathrm{FCmax}}$	$T_{\mathrm{FEmax}}$	${\overset{¯}{T}}_{\mathrm{CL}}$	${\overset{¯}{T}}_{\mathrm{CR}}$	${\overset{¯}{T}}_{C}$	$T_{\mathrm{Cmax}1}$	$T_{\mathrm{CLmax}}$	$T_{\mathrm{CRmax}}$	$T_{\mathrm{Cmax}2}$	$T_{\mathrm{CEmax}}$	$T_{\mathrm{Mmax}}$	$T_{\max}$
IRT-1	0.82	0.79	0.82	0.80	0.81	0.84	0.86	0.88	0.87	0.88	0.88	0.94	0.93	0.94	0.95	0.89	0.95
IRT-2	0.82	0.79	0.82	0.79	0.79	0.84	0.87	0.91	0.87	0.90	0.92	0.95	0.93	0.94	0.95	0.88	0.97

The statistical significance of AUC differences between $T_{\mathrm{Cmax}1}$, $T_{\mathrm{FEmax}}$, $T_{\mathrm{CEmax}}$, and $T_{\max}$ was evaluated using six pairwise tests (Table 4). For both IRTs, there was no statistically significant difference (p≥0.05) between AUC values of $T_{\max}$ and $T_{\mathrm{CEmax}}$, and these parameters    were both significantly higher (p<0.05) than $T_{\mathrm{FEmax}}$. When compared to $T_{\mathrm{Cmax}1}$, $T_{\max}$ showed a significantly higher AUC, but $T_{\mathrm{CEmax}}$ showed a significantly greater result for only one of the two IRTs. Overall, this AUC comparison indicated that $T_{\max}$ and $T_{\mathrm{CEmax}}$ should provide the most optimal results when used for fever screening.

**Table 4:** Results of pairwise comparisons of the AUC values for $T_{\max}$, $T_{\mathrm{CEmax}}$, $T_{\mathrm{FEmax}}$, and $T_{\mathrm{Cmax}1}$.

	IRT-1			IRT-2		
	AUC difference	AUC difference with 95% CI	p-value	AUC difference	AUC difference with 95% CI	p-value
$T_{\max}-T_{\mathrm{FEmax}}$	0.095	0.036 to 0.154	0.002	0.083	0.024 to 0.141	0.006
$T_{\mathrm{CEmax}\;}-T_{\mathrm{FEmax}}$	0.095	0.031 to 0.159	0.003	0.096	0.034 to 0.158	0.002
$T_{\max}-T_{\mathrm{Cmax}1}$	0.067	0.005 to 0.129	0.034	0.052	0.020 to 0.10	0.016
$T_{\mathrm{CEmax}\;}\;-T_{\mathrm{Cmax}1}\;$	0.068	0.009 to 0.126	0.023	0.038	−0.003 to 0.079	**0.068**
$T_{\mathrm{Cmax}1}\;-T_{\mathrm{FEmax}}\;$	0.028	−0.042 to 0.098	**0.437**	0.044	−0.022 to 0.111	**0.192**
$T_{\max}-T_{\mathrm{CEmax}}$	0.000	−0.018 to 0.019	**0.975**	0.013	−0.008 to 0.034	**0.214**

Note: Bold values indicate p>0.05. CI means confidence interval.

Facial temperatures measured by IRTs are typically at least 1°C to 2°C lower than $T_{\mathrm{ref}}$.^34^ Therefore, it is necessary to either calibrate $T_{\mathrm{IRT}}$ to $T_{\mathrm{ref}}$ or find a suitable cutoff temperature for each IRT during fever screening. For consistency with prior IRT studies,^18^^,^^20^ we used the latter method. Our results above (Fig. 6 and Table 4) were based on defining fever as $T_{\mathrm{ref}}\geq 37.5{}^{\circ}C$, from which an optimal cutoff temperature can be obtained for each IRT (first row in Tables 5–7). We also calculated the optimal cutoff temperature under diagnostic thresholds of 37.8°C and 38°C.

**Table 5:** Optimal IRT cutoff temperatures and related Se/Sp values for $T_{\max}$ under different $T_{\mathrm{ref}}$ thresholds.

Oral thermometer	IRT-1				IRT-2			
$T_{\mathrm{ref}}$ diagnostic thresholds (°C)	Actual febrile #	IRT cutoff temperature (°C)	Se	Sp	Actual febrile #	IRT cutoff temperature (°C)	Se	Sp
37.5	47	36.19	0.85	0.94	47	36.11	0.94	0.89
37.8	30	36.28	0.93	0.94	30	36.38	0.93	0.95
38.0	19	36.29	1.00	0.93	19	36.87	1.00	0.98

**Table 6:** Optimal IRT cutoff temperatures and related Se/Sp values for $T_{\mathrm{CEmax}}$ under different $T_{\mathrm{ref}}$ thresholds.

Oral thermometer	IRT-1				IRT-2			
$T_{\mathrm{ref}}$ diagnostic thresholds (°C)	Actual febrile #	IRT cutoff temperature (°C)	Se	Sp	Actual febrile #	IRT cutoff temperature (°C)	Se	Sp
37.5	47	35.96	0.89	0.87	47	36.11	0.87	0.92
37.8	30	36.05	0.93	0.90	30	36.25	0.93	0.95
38.0	19	36.28	1.00	0.95	19	36.58	1.00	0.97

**Table 7:** Optimal IRT cutoff temperatures and related Se/Sp values for $T_{\mathrm{Cmax}1}$ under different $T_{\mathrm{ref}}$ thresholds.

Oral thermometer	IRT-1				IRT-2			
$T_{\mathrm{ref}}$ diagnostic thresholds (°C)	Actual febrile #	IRT cutoff temperature (°C)	Se	Sp	Actual febrile #	IRT cutoff temperature (°C)	Se	Sp
37.5	47	35.22	0.85	0.79	47	35.37	0.83	0.88
37.8	30	35.32	0.80	0.84	30	35.41	0.87	0.87
38.0	19	35.76	0.84	0.96	19	35.94	0.89	0.97

According to our results, $T_{\max}$ and $T_{\mathrm{CEmax}}$ provided the best approaches for fever detection and did not exhibit significant differences in performance. However, the approach that most closely adheres to the recommendations in ISO/TR 13154 is $T_{\mathrm{Cmax}1}$. Therefore, we used all three temperatures in evaluating the optimal Se and Sp for different $T_{\mathrm{ref}}$ diagnostic thresholds in Tables 5–7. Cutoff temperatures were calculated to optimize Se and Sp simultaneously.^38^

These results further illustrate how diagnostic performance decreased from $T_{\max}$ to $T_{\mathrm{CEmax}}$ to $T_{\mathrm{Cmax}1}$. The Se/Sp values for $T_{\max}$ and $T_{\mathrm{CEmax}}$ are similar, which reflects the extent of overlap in their ROC curves (Fig. 6) and minimal AUC differences (Table 4). Performance was good for $T_{\mathrm{Cmax}1}$, yet significantly lower than for $T_{\max}$ and $T_{\mathrm{CEmax}}$. While discrimination of febrile subjects was superior for a cutoff threshold of 38°C, impressive outcomes were also obtained for lower grade fever thresholds at 37.5°C and 37.8°C. The trend of increasing Se/Sp with diagnostic threshold may be due to a larger difference between the temperature of febrile subjects and the normal temperature value where most of the subjects were; a normal temperature with some error is less likely to reach 38°C.

### 3.4 Blackbody Compensation

Temperature compensation using a validated BB was recommended by ISO/TR 13154 to improve IRT system stability. To quantify the impact of this compensation, correlation coefficients between the facial temperatures and $T_{\mathrm{ref}}$ (Table 8) and the AUC values based on a diagnostic threshold of 37.5°C (Table 9) were calculated without BB compensation. Comparison of Table 2 with Table 8 shows that the r values with BB compensation increased for all facial temperatures measured by both IRTs. The increases for $T_{\mathrm{Cmax}1}$, $T_{\mathrm{FEmax}}$, $T_{\mathrm{CEmax}}$, and $T_{\max}$ are 7%, 13%, 12%, and 12% for IRT-1, respectively, and 5%, 4%, 6%, and 5% for IRT-2, respectively. Comparison of Table 3 with Table 9 shows that the AUC values with BB compensation also increased for all facial temperatures. The increases for $T_{\mathrm{Cmax}1}$, $T_{\mathrm{FEmax}}$, $T_{\mathrm{CEmax}}$, and $T_{\max}$ are 2%, 3%, 4%, and 4% for IRT-1, respectively, and 2%, 1%, 3%, and 3% for IRT-2, respectively.

**Table 8:** Pearson correlation coefficients (r values) between facial temperatures and $T_{\mathrm{ref}}$ (no BB compensation).

	Forehead							Inner canthi								Mouth	Face
	$T_{\mathrm{FC}}$	$T_{\mathrm{FT}}$	$T_{\mathrm{FB}}$	$T_{\mathrm{FL}}$	$T_{\mathrm{FR}}$	$T_{\mathrm{FCmax}}$	$T_{\mathrm{FEmax}}$	${\overset{¯}{T}}_{\mathrm{CL}}$	${\overset{¯}{T}}_{\mathrm{CR}}$	${\overset{¯}{T}}_{C}$	$T_{\mathrm{Cmax}1}$	$T_{\mathrm{CLmax}}$	$T_{\mathrm{CRmax}}$	$T_{\mathrm{Cmax}2}$	$T_{\mathrm{CEmax}}$	$T_{\mathrm{Mmax}}$	$T_{\max}$
IRT-1	0.44	0.41	0.47	0.46	0.42	0.50	0.55	0.57	0.57	0.60	0.60	0.64	0.65	0.66	0.67	0.59	0.70
IRT-2	0.45	0.38	0.48	0.45	0.39	0.52	0.60	0.51	0.48	0.53	0.56	0.67	0.66	0.69	0.71	0.58	0.75

**Table 9:** AUC values from the ROC curves of different facial temperatures (no BB compensation).

	Forehead							Inner canthi								Mouth	Face
	$T_{\mathrm{FC}}$	$T_{\mathrm{FT}}$	$T_{\mathrm{FB}}$	$T_{\mathrm{FL}}$	$T_{\mathrm{FR}}$	$T_{\mathrm{FCmax}}$	$T_{\mathrm{FEmax}}$	${\overset{¯}{T}}_{\mathrm{CL}}$	${\overset{¯}{T}}_{\mathrm{CR}}$	${\overset{¯}{T}}_{C}$	$T_{\mathrm{Cmax}1}$	$T_{\mathrm{CLmax}}$	$T_{\mathrm{CRmax}}$	$T_{\mathrm{Cmax}2}$	$T_{\mathrm{CEmax}}$	$T_{\mathrm{Mmax}}$	$T_{\max}$
IRT-1	0.79	0.78	0.80	0.78	0.77	0.80	0.83	0.87	0.85	0.87	0.87	0.91	0.89	0.91	0.91	0.88	0.92
IRT-2	0.81	0.79	0.81	0.78	0.78	0.84	0.87	0.89	0.85	0.88	0.90	0.93	0.91	0.93	0.93	0.86	0.94

## 4 Discussion

In an extensive clinical study, we have evaluated the use of IRTs under standardized conditions and collected a wide range of data on facial temperatures and their correlation to oral measurements. These data have yielded valuable insights into IRT-based temperature estimation and fever detection capabilities and the factors that impact system performance.

### 4.1 Thermographic Screening Accuracy and Standardization

This study was largely based on two international consensus documents described above—IEC 80601-2-59 and ISO/TR 13154.^23^^,^^24^ The guidance provided by these publications helped ensure that the devices used in this research had a high level of image quality and that the acquisition methods—including instructions to subjects—were optimized to enable accurate measurements. The optimal approaches identified in our study produced results that were equal to or better than most prior relevant works in terms of absolute agreement with, and correlation to, reference measurements, as well as discrimination of febrile subjects.

Our findings showed that the differences between $T_{\mathrm{ref}}$ and temperatures of different facial regions were in the ranges of 1.6°C to 2.8°C for the forehead region, 1.4°C to 2.4°C for the inner canthi regions, 1.7°C to 1.8°C for the mouth region, and 1.2°C to 1.3°C for the maximum face temperature. The magnitude of these results is smaller than results from Nguyen et al.,^17^ who showed differences in the range of 2.1°C to 8.7°C between $T_{\mathrm{ref}}$ and facial maximum temperature by three IRTs; similarly Chan et al.^18^ showed forehead temperatures differences of 3°C and 3.9°C for febrile and afebrile subjects, respectively. Our results also showed strong correlations between IRT-measured temperatures ($T_{\mathrm{IRT}}$) and $T_{\mathrm{ref}}$, with both IRTs producing r values as high as the 0.75 to 0.80 range. These values are much higher than several prior studies that found r values between IRT and oral temperatures of no greater than 0.45.^2^^,^^17^^,^^39^ Scatter plots of $T_{\mathrm{IRT}}$ versus $T_{\mathrm{ref}}$ provided in prior studies, such as Chan et al.,^18^ also do not show the strong linear trends seen in our $T_{\max}$ and $T_{\mathrm{CEmax}}$ data (Fig. 5). It is likely that this improvement in correlation is due to control methods that help to reduce measurement variability, including stability correction with a BB, reduction of confounding environmental factors, multiframe averaging, and the use of canthi regions and full-face maximums in thermal images.

Strong temperature correlations enabled discrimination between febrile and afebrile subjects to a high degree of accuracy. For a low-grade fever diagnostic threshold of 37.5°C, $T_{\max}$ data produced an AUC value of 0.95 to 0.97 and Se/Sp values in the 0.85 to 0.95 range. For a diagnostic threshold of 37.8°C, Se/Sp values increased to the 0.93 to 0.95 range. These results for relatively low-grade fever detection, as well as findings at higher diagnostic thresholds shown in Table 5, compare favorably with the literature. In a study of airport travelers, Priest et al.^20^ found an AUC of 0.71 (Se/Sp=0.86/0.71) for a fever threshold of 37.5°C using full-face maximum temperatures. Nishiura and Kamiya^2^ estimated that the AUC values were 0.79 and 0.75 for threshold temperatures of 37.5°C and 38.0°C, respectively. Nguyen et al.^17^ compared IRT performance for fever screening using images of the face and neck, with 37.8°C as the fever threshold. In this study, AUC values of 0.96 and 0.92 were found for two IRTs, yet the corresponding r values of 0.43 and 0.42 do not appear sufficient for high accuracy measurements. Hewlett et al.^19^ obtained AUC values of 0.86 and 0.90 for fever thresholds of 37.8°C and 38°C, respectively, but did not report r values or results for 37.5°C. These comparisons provide substantial evidence that an approach based largely on adherence to recently published standards has the potential to advance IRT-based fever-screening capability.

### 4.2 Comparison of Facial Temperatures

The 17 facial temperatures extracted from each subject’s thermal image can be categorized by facial region (forehead, canthi, mouth, and entire face) or by measurement location selection method (fixed location versus maximum value of a defined region). Analyzing our extensive clinical testing results provided insight into key trends and potential approaches for optimizing IRT-based fever screening.

IRT system performance was highly dependent on measurement location, with the forehead producing lower accuracy than canthi regions. Temperatures determined from five fixed locations on the forehead ($T_{\mathrm{FC}}$, $T_{\mathrm{FT}}$, $T_{\mathrm{FB}}$, $T_{\mathrm{FL}}$, and $T_{\mathrm{FR}}$) had relatively low correlations (r<0.50) with $T_{\mathrm{ref}}$ and larger pairwise differences. Fixed locations in the canthi region showed moderately strong correlations (r values of 0.51 to 0.63) with $T_{\mathrm{ref}}$ and their pairwise differences from $T_{\mathrm{ref}}$ were also relatively small. Similarly, the maximum-value data for canthi regions showed better performance than the forehead or mouth regions. This result aligns with a prior comparison of IRT-based eye and forehead measurements.^43^ In our study, maximum value of the entire face ($T_{\max}$) provided better performance than the forehead ($T_{\mathrm{FEmax}}$) in terms of correlation and fever detection; this finding is consistent with a prior study that compared maximum temperatures from the full face and forehead (r values of 0.43 and 0.36, respectively).^18^ Differences in performance between the forehead and inner canthi are likely due to perfusion of the canthi from the internal carotid (ophthalmic) artery, proximity to large vessels, and relatively thin skin,^32^ whereas the forehead is less diffusely perfused and more susceptible to convective and evaporative cooling.^43^^,^^44^ These findings may shed light on the poor sensitivity values found in some NCIT studies.^14^

Overall, the maximum value in a region showed better diagnostic performance and correlation with $T_{\mathrm{ref}}$ than the value at a fixed location within this region, with greatest r values and AUC values for $T_{\max}$, followed by $T_{\mathrm{CEmax}}$ and then $T_{\mathrm{FEmax}}$. Prior studies have also found that maximum-values approaches tended to provide greater performance.^18^ $T_{\mathrm{CEmax}}$ and $T_{\max}$ yielded similar r values and statistically equivalent AUC values, as well as significantly higher AUC values than $T_{\mathrm{FEmax}}$. Interestingly, Fig. 5 shows that unlike the relatively tight cluster of normal-range data points ($T_{\mathrm{ref}}=36.4{}^{\circ}C$ to 37.4°C), data for $T_{\mathrm{Cmax}1}$ exhibit a tail extending to lower IRT-measured values than other datasets. This feature is also present in the few scatter plots that have been published from clinical IRT data.^18^^,^^43^ Additionally, we found that individual hairs on the forehead degraded accuracy. The improved performance observed for maximum region temperatures may be due in part to subject-to-subject variations in facial anatomy and physiology that cause unpredictable nonuniformity in spatial temperature distribution. Taking the maximum temperature of a region affords greater robustness to such variations.

As noted above, approaches involving the inner canthi or maximum-temperature locations provided higher levels of performance. Therefore, it is not surprising that $T_{\mathrm{CEmax}}$—which involves both of these features—provided one of the best options of the 17 temperatures tested. The finding that $T_{\max}$ provided slightly better performance than $T_{\mathrm{CEmax}}$ is a more unexpected result, because it was not advocated in ISO/TR 13154 as a “robust measurement site,” as the inner canthi were. However, this approach has been used in a number of prior studies^17^^,^^18^^,^^20^ likely due to its combination of simplicity and effectiveness. These prior studies achieved relatively high-Se/Sp values (0.7 to 0.9) using this approach. In part, this effectiveness stems from the fact that the inner canthi are a key thermal feature in full-face images, as discussed Sec. 4.3. In spite of these benefits, there may be unresolved challenges related to the use of $T_{\max}$, such as confounding physiological factors (e.g., sinusitis) that impact temperature distributions.^24^

### 4.3 Distribution of Thermal Maxima in Full-Face Images

**Fig. 7:**
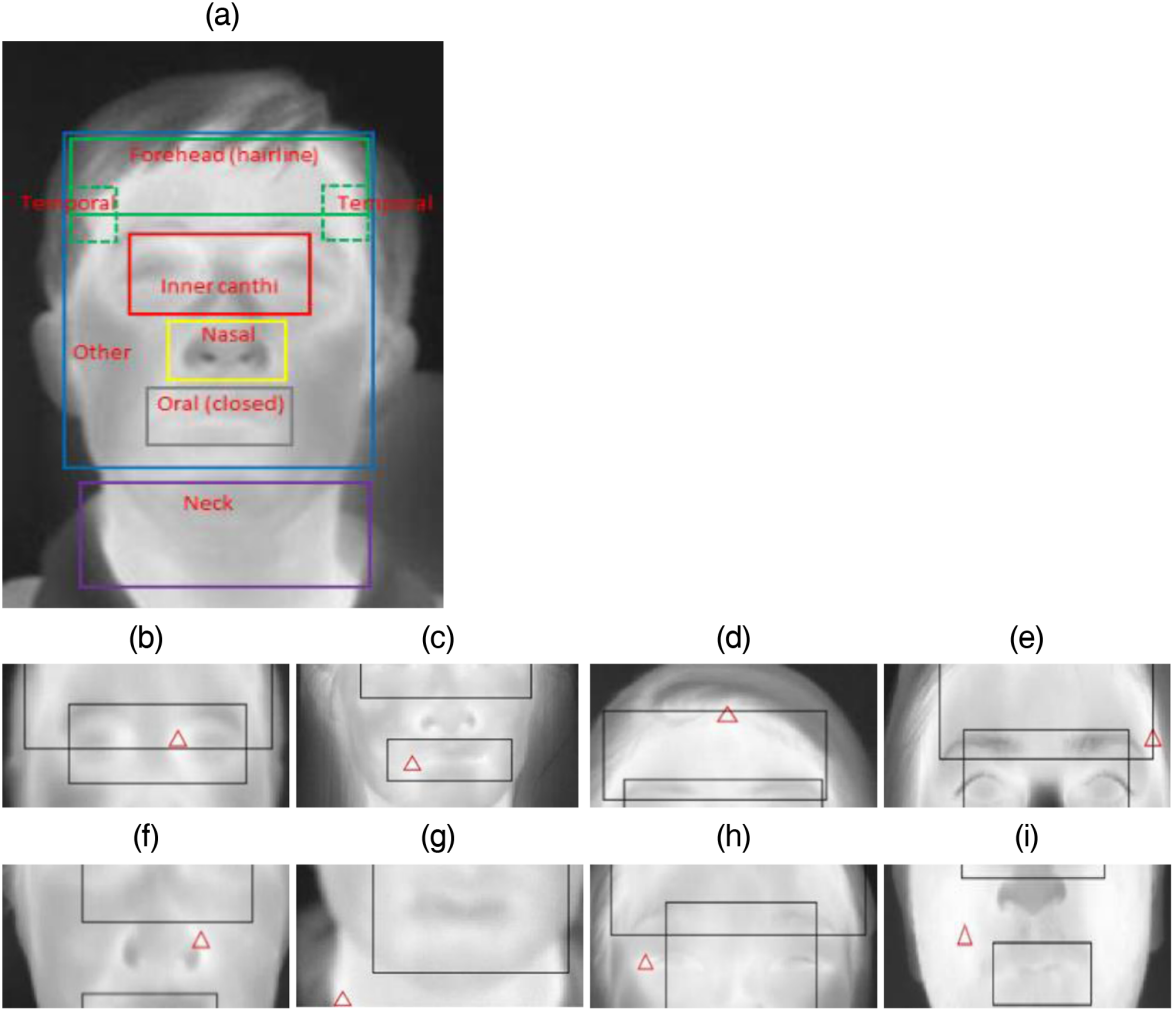
Thermal images illustrating (a) delineation of regions for thermal maxima analysis and (b)–(i) examples of maxima in human subjects. (a) Defined regions, (b) inner canthi, (c) oral (closed), (d) forehead (hairline), (e) temporal, (f) nasal, (g) neck, (h) other (outer canthus), and (i) other (cheek). Photo (a) is of author Q. Wang.

To better understand the results obtained with $T_{\max}$, we evaluated the distribution of locations where maximum temperatures occurred over 3252 thermal images collected by the two IRTs from the first round of measurements. The locations of thermal maxima in full-face images are summarized in Fig. 7 and Table 10. According to Table 10, thermal maxima appeared most commonly (59.5%) in the inner canthi region, followed by oral (21.7%), forehead (8.8%), nasal (4.1%), and temporal (3.6%) regions. The predominance of inner canthi maxima is expected given what is known regarding perfusion in this region. A relatively large fraction of maxima occurred in the oral region, likely due to perfusion from the facial artery which is closer to the external carotid artery than the vessels that perfuse most facial regions. The forehead maximum was typically along the hairline, likely due to the thermal insulation effect of hair. Some thermal maxima appeared in the temporal region, likely due to the superficial temporal arteries. It was unexpected to find maxima in the nasal/nostril region (bottom); whether these are due to some pathologies, such as sinusitis,^24^^,^^45^ or perhaps exhalation of warm air is not currently known.

**Table 10:** Spatial distribution of facial temperature maxima.

Region	Number	%
Inner canthi	645	59.5
Oral (closed)	235	21.7
Forehead (hairline)	95	8.8
Nasal	44	4.1
Temporal	39	3.6
Neck	17	1.6
Other	9	0.8

### 4.4 Quality of a Thermographic Screening System

The IEC 80601-2-59 standard^23^ defines an ST as a system composed of an IRT and an external temperature reference source (usually a BB with known temperature and emissivity), and in some cases, a computer and software for data acquisition, processing, and storage. Therefore, most results in this paper, except for the data in Sec. 3.4, are technically not results of two thermal cameras, IRT-1, and IRT-2, rather, two fever-screening systems, (IRT-1 + BB) and (IRT-2 + BB). We have evaluated these two systems in our previous work^28^ and found that their uniformity, stability, drift, minimum resolvable temperature difference, and laboratory accuracy all satisfied the standard requirements.

The use of a BB for temperature compensation had a moderate impact on IRT screening ability. In our prior study,^28^ such compensation vastly improved the stability of IRT-2 and enabled the system to meet IEC 80601-2-59 performance specifications. Except to measure and compensate for long-term drift, the use of three-frame averaging in the current study may have improved stability to the point where the BB was no longer critical. If no frame averaging is used, the use of a BB would likely be more critical for fever detection. Additionally, the current study was executed in an environment with relatively stable ambient temperature; it is likely that in a less controlled screening location with larger, more rapid thermal fluctuations, BB compensation would be more important.

While inherent IRT instrumentation quality is critical, the performance also depends on effective implementation. The use of control methods, such as an absorbing background, multiple frame imaging, and thermally stable, forward-facing subjects also likely helps to optimize screening performance. Given that many of these confounding factors have been addressed in our study, the results presented here likely indicate a best-case performance level. As control, if methods we have implemented are removed—which may be necessary in certain real-world screening situations—it is likely that performance will degrade. The degree to which removal of any specific control will impact results is beyond the scope of the current study but may be important for predicting real-world performance.

### 4.5 Fever Screening During an Epidemic

The primary purpose of this study was to facilitate the implementation of IRT systems and practices that enable optimal measurement accuracy and highly effective fever screening during epidemics. However, achieving effective screening can be a complex process, as many factors need to be addressed beyond the physics, instrumentation, and acquisition procedures. While our results showed that some facial temperatures had good discrimination abilities with high AUC values, some previous literature claimed that thermography was not highly effective for fever screening during disease outbreaks.^1^^,^^2^^,^^46^^,^^47^ This may have been due to device instability,^7^^,^^11^^,^^32^^,^^34^^,^^48^ inappropriate temperature reading locations, nonstandard calibration, and environmental controls.^28^ In a future study, we will address the impact of environmental conditions outside those that the standard recommends—such as higher or lower ambient temperatures that might be encountered near the entry of a building—on IRT accuracy and linearity.

The frequency at which fever presents as a symptom is another impediment to successful screening. In the current COVID-19 outbreak, many of those infected are largely asymptomatic and only 73% have exhibited a fever;^3^ in 2009, only half of H1N1 outbreak cases had temperatures of ≥37.8°C;^8^ and a 2011 study indicated that none of the 30 subjects identified as being flu-infected had a temperature of 37.8°C or greater, and only two had a temperature of 37.5°C.^20^ Therefore, while IRT-based screening can detect individuals with elevated body temperature, it is not a viable stand-alone tool for screening for individuals infected with specific diseases.^49^ It may play an adjunct role along with other screening evaluations. Since fever is only one common symptom of infectious disease, an effective screening process should include evaluation of a range of symptoms.^3^^,^^7^^,^^8^ The future development of an integrative screening system may include thermography along with other optical imaging approaches for evaluation of vital signs, such as pulse rate and respiratory rate, and other physiological parameters.^8^

Given that $T_{\mathrm{CEmax}}$ and $T_{\max}$ provided the best performance, it is worth considering issues that might influence the decision to implement one approach or the other. Acquiring a full-face region for calculation of $T_{\max}$ would likely be easier to accomplish and performed more reliably than determining $T_{\mathrm{CEmax}}$ via auxiliary visible-light imaging and computationally intensive techniques for coregistration of inner canthi regions.^37^ This may be particularly important in a high-throughput situation where delays due to computer processing or image coregistration errors could become highly inconvenient. However, implementing an approach that blindly determines the maximum temperature from a full-face thermal image may increase the need to identify confounding pathological/physiological conditions such as sinusitis.^24^^,^^45^^,^^50^ To accomplish this task rapidly and effectively may require significant screener training, although automated approaches (e.g., deep-learning algorithms) could also be developed to augment or replace manual assessments.

Another practical challenge involves the identification of an appropriate reference temperature diagnostic threshold, given the diversity of values that have been implemented. The Centers for Disease Control and Prevention (CDC) has recommended the use of 38°C,^51^ whereas prior human subject studies have been based on 37.5°C,^7^^,^^39^^,^^40^ 37.6°C,^52^ 37.7°C,^32^^,^^48^ 37.8°C,^17^^,^^19^^,^^20^ and 38°C.^35^ Different thresholds have been used for different outbreaks, such as ≥38°C for SARS^1^ and 37.7°C for adults and 37.9°C for children in an H1N1 study.^8^ The literature indicates that as the threshold temperature decreases, diagnostic accuracy typically degrades. Uncertainty in normal body temperatures—which can be influenced by circadian rhythm, age, physical exertion, and other factors—can further increase error in screening tasks.^15^^,^^35^^,^^39^^,^^53^^–^^56^ For example, studies have shown that the core body temperature in the morning may be significantly lower than in the afternoon.^53^^–^^55^ Additionally, a recent study indicates that normal body temperature has decreased on average since the establishment of the 37°C threshold 150 years ago.^57^ In spite of these obstacles, our results indicated that IRT systems are capable of detecting low-grade fever (37.5°C) in subjects, which could mean that early-stage infections and those producing only moderate symptoms could be more readily identified. The significance of this ability is demonstrated by the fact that of the subjects with $T_{\mathrm{ref}}$ values over 37.5°C in our study, 60% would not have exceeded the CDC recommended diagnostic threshold of 38°C.

Even if a suitable diagnostic threshold for fever based on body temperature can be defined, determining the IRT cutoff temperature for fever screening requires a variety of considerations. While we calculated optimal cutoff temperature to optimize both Se and Sp, this may not represent an optimal value for real-world use. For a severe disease, lower cutoff values may be needed to minimize false negatives in primary screening. Given the typically low prevalence of diseased individuals in a screening population, the false positive rate in primary screening will be high (and thus the positive predictive value low). On the other hand, it may also be important to balance the burden on the population being screened (e.g., travel delays) and screening personnel (e.g., workload, fatigue, and cost to health agencies).^17^^,^^39^

## 5 Conclusions

Overall, our clinical study results support the conclusion that adherence to international consensus guidelines regarding IRT system specifications and implementation contributes to optimization of measurement accuracy and detection of febrile individuals. These guidelines include environmental controls as well as subject preparations and acclimation prior to measurements. Two additional findings were made: first, targeted measurement of a small inner canthi area may be unnecessary, with full-face maximum temperatures providing the best performance followed closely by the maximum of a wider inner canthi region. Second, while temperature compensation with a high-quality BB can improve IRT system performance, the impact was modest on our high-quality IRTs and highly controlled setup, which incorporated multiframe averaging. Our findings also indicated that forehead temperatures provide generally inferior estimation of reference (oral) temperature relative to the inner canthi and full-face maximum temperature. The optimal approaches identified here achieved correlation coefficients of ∼0.75 and AUC values of ∼0.95 for detection of low-grade fever (37.5°C). This capacity may facilitate detection of a wider range of disease presentations than a less accurate method would allow. Future work will involve further analysis of our clinical study results to address the impact of confounding factors relating to intersubject and environmental variability.
